# miRNAs as Biomarkers and Possible Therapeutic Strategies in Rheumatoid Arthritis

**DOI:** 10.3390/cells11030452

**Published:** 2022-01-28

**Authors:** Tomasz Kmiołek, Agnieszka Paradowska-Gorycka

**Affiliations:** Department of Molecular Biology, National Institute of Geriatrics, Rheumatology and Rehabilitation, 02-637 Warsaw, Poland; paradowska_aga@interia.pl

**Keywords:** rheumatoid arthritis (RA), immune and inflammatory responses, bone destruction, synovial hyperplasia, microRNA, autoimmune disease

## Abstract

Within the past years, more and more attention has been devoted to the epigenetic dysregulation that provides an additional window for understanding the possible mechanisms involved in the pathogenesis of autoimmune rheumatic diseases. Rheumatoid arthritis (RA) is a heterogeneous disease where a specific immunologic and genetic/epigenetic background is responsible for disease manifestations and course. In this field, microRNAs (miRNA; miR) are being identified as key regulators of immune cell development and function. The identification of disease-associated miRNAs will introduce us to the post-genomic era, providing the real probability of manipulating the genetic impact of autoimmune diseases. Thereby, different miRNAs may be good candidates for biomarkers in disease diagnosis, prognosis, treatment and other clinical applications. Here, we outline not only the role of miRNAs in immune and inflammatory responses in RA, but also present miRNAs as diagnostic/prognostic biomarkers. Research into miRNAs is still in its infancy; however, investigation into these novel biomarkers could progress the use of personalized medicine in RA treatment. Finally, we discussed the possibility of miRNA-based therapy in RA patients, which holds promise, given major advances in the therapy of patients with inflammatory arthritis.

## 1. Introduction

Rheumatoid arthritis (RA) is a chronic, systemic inflammatory autoimmune disease that affects 1% of the population worldwide [1]. RA is a molecularly heterogeneous, complex, multifactorial disease with different biological and clinical characteristics. The chronic inflammatory process and autoimmunity associated with the disease mostly affect the synovium, which leads to joint damage and synovitis through the infiltration of inflammatory cells into the tissues [2]. A number of etiological factors associated with RA have been identified as hereditary, genetic, environmental and lifestyle factors. Epigenetic mechanisms may serve as a dynamic link between environment, genotype and phenotype. Accumulating evidence has demonstrated that aberrant epigenetic modification plays a pivotal role in triggering the dysregulation of T cell activation, leading to the incidence of RA [3,4,5]. Within the epigenetic factors participating in RA, micro-RNAs (miRNAs), playing an important role in many biological processes, are considered to be potential therapeutic targets for many autoimmune/inflammatory diseases, including RA [6,7]. Altered miRNA expression has been associated with the enhanced secretion of proinflammatory cytokines and increased numbers of inflammatory signaling pathways, as well as other processes that maintain the vicious cycle of autoimmunity [8]. Moreover, miRNAs also show immense potential for not only diagnostic (circulating miRNAs in serum are attractive biomarkers) but also therapeutic applications.

The treatment of RA is still a significant clinical challenge. Traditional treatments for RA involve suppressing the excessive immune and inflammatory responses, which may only help to relieve the symptoms of RA and delay the progression of the disease, not cure it. Moreover, these strategies lead to several systemic side effects. The therapy of RA is carried out based on targeted synthetic disease-modifying antirheumatic drugs (DMARDs), glucocorticoids and conventional synthetic DMARDs (csDMARDs), as well as biological DMARDs (bDMARDS). The csDMARDs used in RA treatment include methotrexate (MTX), hydroxychloroquine, sulfasalazine and leflunomide. MTX is the first choice regardless of disease activity [9], despite the fact that the ACR guidelines favor hydroxychloroquine and sulfasalazine over MTX for patients with lower disease activity to reduce the negative effects of MTX [10,11]. At the present time, among the bDMARDs used in the treatment of RA, we can distinguish anti-TNF drugs (such as etanercept (ETA), adalimumab (ADA), infliximab (IFX), certolizumab pegol, golimumab (GOL)), and TNF inhibitors are commonly used in the standard treatment of RA, mainly for patients in whom MTX treatment was unsuccessful. Around 80% of RA patients receive mixed therapy with MTX and TNF inhibitors [12,13]. Next, treatments use IL-6 receptor antagonists (tocilizumab (TCZ), sarilumab) and recombinant IL-1R blockers (anakinra). TCZ and sarilumab bind to the transmembrane IL-6 receptor, blocking the signaling of IL-6 [14]. Recent clinical trials show that sarilumab is more efficient in RA patients who do not respond well to MTX or TNF inhibitors [15]. Janus kinase (JAK) inhibitors are another group of inhibitors that are used in the treatment of RA. JAK is a receptor-associated tyrosine kinase whose role is to intervene in signals starting from inside a cell through transcription factors, such as STAT [16]. The JAK family consists of JAK1, JAK2, JAK3 and tyrosine kinase 2 (Tyk2). Baricitinib and tofacitinib are drugs already used in the treatment of RA. Additionally, they are JAK inhibitors. Baricitinib selectively inhibits JAK1 and JAK2, whereas tofacitinib inhibits JAK1, JAK2, JAK3 and Tyk2 [17,18]. Clinical trials show baricitinib presented an improvement of RA symptoms for subjects who did not respond well towards MTX, TNF inhibitors or csDMARDs [19,20,21]. A new strategy of treatment for RA should consist of the co-stimulation of T cell blockers using abatacept, which is an anti-CD80/86 inhibitor, B cell depletion drugs, such as rituximab, which is a chimeric anti-CD20 mAb, and JAK, using tofacitinib or baricitinib, which belong to the targeted synthetic DMARDS (tsDMARDs) [22,23,24,25,26].

Therefore, research investigating the effective molecular targets for treating RA is underway. The next, very important step in the near future will be to utilize miRNA as a novel therapy for RA. Various miRNAs related to inflammatory cytokines, osteoclast differentiation and synovial cell proliferation have been investigated for possible application in RA treatment [27]. Noting the importance of novel therapies, researchers focus on looking for reliable predictive biomarkers for better prognosis and therapeutic responses [28]. Researchers hope that miRNA will facilitate faster disease detection and better and more precise therapy.

## 2. Characteristics and Pathology of RA

RA is a chronic, systemic inflammatory autoimmune disease. The most frequent symptoms of RA are pain, stiffness and tenderness of joints, which lead to systemic complications, progressive disability and even early death. Current treatment relies on aggressive therapy immediately following diagnosis, with the pursuit of clinical remission by increasing therapy, depending on disease activity [1]. The main characteristics of RA are synovial hyperplasia and inflammation, the deformity of cartilage and bone, the production of anti-citrullinated protein antibodies (ACPA) or rheumatoid factors (RF), which are autoantibodies typical for RA, and systemic features, such as skeletal, pulmonary, cardiovascular and psychological disorders [29]. Additionally, RA rarely goes into total remission, and requires ongoing pharmacological therapy [9,30,31,32].

In RA, inflammation leads to bone destruction, cartilage degradation and synovial inflammation. Bone destruction is characterized by a disruption in bone formation. Macrophages activated by CD4 T Helper cells (Th cells) produce proinflammatory cytokines, such as tumor necrosis factor (TNF)-α, interleukin (IL)-1 and IL-6, which activate osteoclastogenesis. These cytokines also activate the production of macrophage colony-stimulating factor (M-CSF) and receptor activator of nuclear factor kappa B ligand (RANKL) from synovial joint cells, and activate RANKL on osteoblast cells to bind with receptor RANK on osteoclast precursor cells for osteoclast maturation [33]. In contrast, osteoclastogenesis is inhibited by osteoprotegerin, which blocks the osteoclast differentiation pathway by inactivating RANKL. In consequence, mature osteoclasts are responsible for the degradation of osteonectin and aggrecan, causing chronic joint destruction [34].

**Figure 1:**
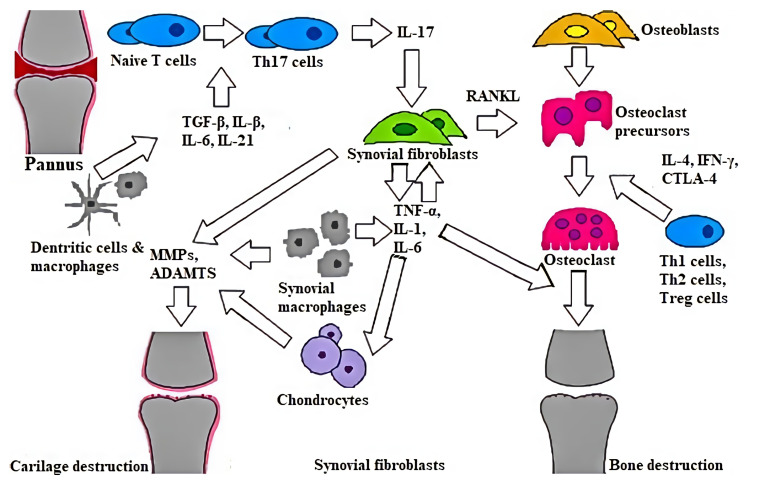
The pathology of rheumatoid arthritis (RA), showing the process of cartilage and bone destruction. RA is distinguished by the formation of pannus, which is an abnormal layer of tissue created over the joint surface, leading to the erosion of articular cartilage and bone. Pannus contains macrophages, which create inflammatory cytokines, such as tumor necrosis factor (TNF)-α, interleukin (IL)-1 and IL-6, activating osteoclasts and resulting in bone destruction. Additionally, present in pannus are T cells, consisting of regulatory T cells (Treg) and T helper cells (Th), which are made up of the Th1, Th2 and Th17 cell subsets. Th17 cells are differentiated from naive T cells by IL-1β, IL-6, IL-21 and TGF-β. Th17 cells create IL-17, which acts on various immune cells to activate inflammation, and induces RANKL in synovial fibroblasts to activate osteoclasts. Th1 cells create IFN-γ, Th2 cells create IL-4 and Treg cells create CTLA-4, which are responsible for regulating osteoclast differentiation. Matrix metalloproteinases (MMPs) and a disintegrin and metalloproteinase with thrombospondin motifs (ADAMTS), which are created by chondrocytes, synovial fibroblasts and synovial macrophages, lead to cartilage destruction.

Proinflammatory cytokines, such as TNF-α, IL-1 and IL-6, are also responsible for stimulating synovial fibroblast cells to emit the cartilage-degrading enzymes, matrix metalloproteinases (MMPs). These enzymes are specific biomarkers for cartilage destruction in RA [35]. MMPs are inhibited and downregulated by tissue inhibitors of metalloproteinases (TIMPs), so the balance of MMPs and TIMPs in cartilage metabolism is important to maintain homeostasis [36]. Synovium is a primary area for inflammation, where a disturbance in immune tolerance leads to RA. Granulated macrophage colony-stimulating factor (GM-CSF), vascular endothelial growth factor (VEGF), IL-6, IL-1β and IL-17 are the cytokines that have a key role in synovial inflammation and the activation of synovial mononuclear cells. Pannus is part of a synovial membrane made of granular tissues that is abundant with osteoclast. Free-roaming cartilage fragments and bone osteophytes in the synovial cavity are primary significant factors in synovial inflammation [37]. In pannus can be found Th cells, which are made up of the Th1, Th2 and Th17 cell subsets, and regulatory T cells (Treg) (Figure 1) [31]. 

Th cells are responsible for initiating the immune response, and Treg cells are responsible for the regulation of the immune response [38]. Th17 and Treg cells arise from the same naïve CD4+ T cells in different cytokine environments [39]. T cells are converted into Th17 cells by IL-1β, IL-6, IL-21 and transforming growth factor-β (TGF-β). Th17 cells produce IL-17 which, by affecting various cells, activates inflammation and induces RANKL, which activates osteoclasts [40]. Cytokines such as IFN-γ, IL-4 and cytotoxic T-lymphocyte-associated protein 4 (CTLA-4), which are created by Th1, Th2 and Treg cells, are responsible for the regulation of osteoclast differentiation. An imbalance between Th17 and Treg cells occurs in RA, and may play a role in the disease progression [31]. Th17 and Treg cells not only show opposite functional properties, but are also characterized by different cell markers: retinoic acid receptor-related orphan receptor variant 2 (RORC2) is a Th17 marker and forkhead box P3 (Foxp3) is a Treg marker [41]. Both Th17 and Treg cells control the proliferation of each other to maintain equilibrium. Pathogenic Th17 cells mediate pannus growth, osteoclastogenesis and synovial neoangiogenesis, while Treg cells function as a suppressor of autoreactive lymphocytes [42]. In recent years, several studies highlighted that miRNAs are important regulators of the immune response [43]. A few miRNAs, such as miR-155 and miR-146a, affect T cells function. As a result, they modulate autoimmune pathogenesis [44,45,46]. Moreover, the increase in miR-146a and miR-155 in peripheral blood mononuclear cells (PBMCs) from RA patients suggests that miRNAs can be involved at different levels in the regulation of RA pathogenesis [47].

## 3. microRNAs and Their Role in Rheumatoid Arthritis Pathogenesis

miRNAs are single-stranded, conserved, non-coding RNAs, 20–22 nucleotides in length. miRNAs have been the topic of many studies in recent times, and more than 2000 miRNAs have been identified to date. With every new study on miRNAs, it becomes more apparent that miRNAs take part in many biological functions, including cell proliferation and apoptosis. miRNAs direct gene expression post-transcriptionally, and control around one third of the genes in the human genome [48]. miRNAs are also responsible for regulating innate and acquired immunity by contributing to the generation of immune cells, including T cells, B cells and dendritic cells. “Changed” miRNAs initiate the creation of excessive autoantibodies and the secretion of inflammatory cytokines, leading to an immune system imbalance. In this way, miRNAs correlate with different autoimmune diseases, including RA [48,49]. A series of miRNAs were found to be uncontrolled in subsets of cells inside the articular compartment of RA patients, leading to the production of proinflammatory cytokines and the activation of leukocytes, which take part in the immunologic component of effector synovial pathology [50]. Numerous miRNAs related to inflammatory cytokines, synovial cell proliferation and osteoclast differentiation have been identified to date, and attempts have been made to use them in RA treatment [51,52]. Focusing on miRNAs will help to advance the treatment strategies for RA.

miRNAs are responsible for the determination of the expression of many genes. They have a critical part in supporting the improvement and function of the immune system, and many are active in the progress of autoimmune diseases. miRNAs such as let-7a, miR-26, miR-146a, miR-146b, miR-150 and miR-155 have been shown to be meaningfully upregulated in IL-17, producing cells leading to Th17 differentiation and promoting RA pathogenesis via IL-17 [53]. Furthermore, miR-146a, miR-155, miR-132 and miR-16 are responsible for the increase in PBMCs seen in patients with RA compared to healthy subjects [47].

The level of miRNA expression depends on the stage of RA development. In patients at an early stage of RA, serum levels of miR-16, miR-146a, miR-155 and miR-223 are lower than in patients with more advanced RA progression [54]. Furthermore, patients with RA in the earlier stage have a notably lower level of miR-16 and miR-223 in serum when compared to healthy controls, and these miRNAs may serve as biomarkers to better differentiate patients at an early stage of RA from healthy subjects [54].

As shown in Table 1, miR-16, miR-103a, miR-132, miR-145, miR-146a, miR-155, miR-221, miR-22 and miR-301a [8,47,55,56,57,58,59] were increased. These changes affect primarily cytokine secretion and disturbances in the balance between Th17 and Treg cells. For example, for miRNA146a, studies in vitro have shown how stimulation with IL-1 and TNF-α leads to the upregulation of miRNA146a in monocytic cell lines, and how this is regulated by nuclear factor kappa-beta (NF-κB). Other studies show that rheumatoid arthritis synovial fibroblast (RASF), when exposed to TNF-α, increased the expression of miRNA146a.

Table 2 presents miR-21, miR-125b and miR-548a, which decreased in patients with RA [60,61,62]. Lin, S. et al. showed that miR-6089 is downregulated in RASF and synovial fibroblast-like synoviocytes. They observed that the overexpression of this miRNA restrained proliferation and promotes apoptosis and, on the other hand, lowers the secretion of inflammatory cytokines in RASF [63]. Many researchers show favorable correlations between TNF-α and PBMC miRNA146a expression in patients with RA [64]. By taking part in the downregulation of IL-1 and TNF-α production, this downregulation in PBMCs could lead to persistent proinflammatory cytokine production in patients with RA. miRNAs can act as a negative regulator for NF-κB, which leads to the downregulation of the protein levels of IRAK1 and TRAF6, which are the main particles downstream of TNF-α and IL-1 signaling [64].

### 3.1. miRNAs Affecting Rheumatoid Arthritis Synovial Fibroblasts

As mentioned earlier, T cells have a crucial role in RA pathogenesis by activating synovial macrophages and RASFs [29]. Modifications in miRNA expression affect many features of RASFs’ tasks [65]. These changes influence intracellular pathways in RASFs by altering miRNA levels. It can be noticed in pathways such as NF-κB [66,67], JAK/STAT [68,69] and wingless/integrated (Wnt) [70], as well as Toll-like receptor 2 (TLRs) [71]. The disruption of miRNA levels impacts the secretion of proinflammatory cytokines and MMPs, and the proliferation and survival or extinction of RASF. Moreover, some authors have also shown that changes in miRNA levels may counteract inflammation, which leads to tissue damage. Changes in miRNA levels in RASFs and what are they targeting are presented in Table 3 and Table 4 [8].

### 3.2. miRNA Affecting Signaling Pathways

**Figure 2:**
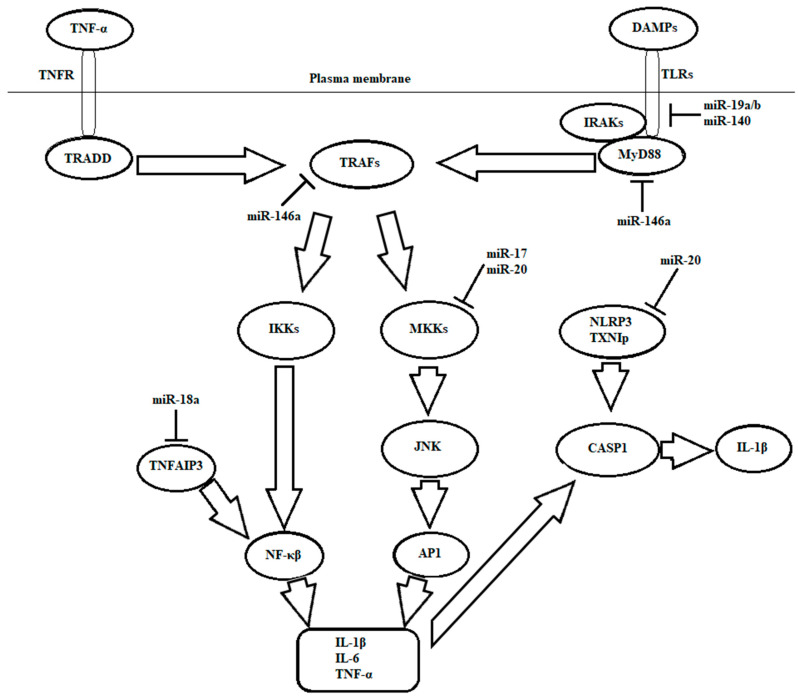
TNF (tumor necrosis factor) and TLR (Toll-like receptor) signaling pathways with the miRNAs that target them.

The role of TNF-α in inflammatory arthritis is well known, and is a starting point for anti-TNF-α therapy. Various miRNAs have been stated to control positively, and others negatively, TNF-α signaling and expression in RA, mainly by targeting the components of the NF-κΒ pathway. miRNA 17-92 cluster in recent years was the topic of many studies, mainly in oncology and its involvement with tumorigenesis, but it was also studied for inflammation in recent times. miRNA 17-92 cluster consists of miR-17, miR-18a, miR-19a/b, miR-20 and miR92 [72]. Earlier studies have proven an anti-inflammatory function of miR-19 and miR-20 in RASFs by modifying TLR and MAPK, which are entailed with the TNF-α pathway. miR-17, by being under-expressed in synovial fibroblasts and the membranes of patients with RA, leads to an anti-inflammatory reaction by affecting MAPK, which is entailed with the TNF-α pathway [73]. miR-20 not only influences the MAPK signaling pathway, but it is also involved within the NLRP3-inflammasome pathway, whereby targeting TXNIP can lower NLRP3 expression [74]. RASF transfected with pre-miR-18 showed proinflammatory phenotypes through the activation of NF-κΒ pathways after stimulation with TNF-α (Figure 2) [75].

Other miRNAs are involved in TNF-α pathway. For example, it has been found that miR-451 overexpression may reduce RASF proliferation as well as reduce proinflammatory cytokines secretion by RASFs. [76]. RASFs with overexpressed miR-125b were found with proinflammatory phenotypes. Additionally, there were higher levels of TNF-α, IL-1β, IL-6 and phospho-p65, which have major roles in the NF-κΒ pathway [66]. Among the previously mentioned miRNAs, miR-146a has been shown to be a regulator of immune and inflammatory replies, as well as being highly expressed in synoviocytes, synovial tissues, PBMCs and the rest of the cells that expressed IL-17 in patients with RA [47,53,77,78,79]. A strong connection was reported between miR-146a and inflammation and the regulation of the proliferation of immune-regulated cells. This beneficial role for miR-146a in inflammatory and immune processes in RA could make it a possible novel target for RA therapy. Additionally, miR-146a has a crucial suppressing role for Treg cells, and the lack of miR-146a in Treg cells can lead to a breach of immunological tolerance [45].

### 3.3. Role of miRNAs in Inflammatory Responses

A few other miRNAs have been shown to be responsible for regulating immune and inflammatory responses in patients with RA; these are presented in Table 5. miR-19a/b was stated to have a negative role in the regulation of inflammation in the human model [80]. It was shown that miR-21 acts by preserving the balance between immune tolerance and activation [81]. The levels of miR-323-3p were discovered to be increased in RASF, and the gene encoding this miRNA can act as a biomarker of the immune and inflammatory response [82]. miR-155 has strong regulatory potential in various immune cells. A positive correlation between miR-155 and inflammation in patients with RA has been found [82], indicating that miR-155 takes part in immune reactions, which results in autoimmune arthritis. miR-155 might be the next novel target for RA therapy [49,83,84,85,86].

### 3.4. Role of miRNAs in Synovial Hyperplasia Responses

The effects of miRNAs on synovial hyperplasia are presented in Table 6. miR-126 overexpression in RASF leads to an increased rate of proliferation and, on the other hand, lower apoptosis. This, in consequence, was related to the reduction of phosphatidylinositol 3-kinase regulatory subunit 2 (PIK3R2) expression and the induction of the PI3K and p-AKT proteins [88,89]. Downregulated miR-221 expression is responsible for the inhibition of the expression of proinflammatory cytokines, as well as for the inhibition of the RASF migration and invasiveness [90]. Niederer F. et al. [86] have demonstrated that the expression level of miR-34a was lower in RASF compared to osteoarthritis (OA) synovial fibroblast. Additionally, miR-34a raised TRAIL-mediated apoptosis in RASFs [86]. miR-15a has been shown to lead to apoptosis through the negative regulation of the expression of B cell lymphoma 2 (Bcl-2), which leads to the suppression of apoptotic processes [91]. Another noteworthy miRNA that impacts synovial hyperplasia is miR-124a, which suppresses the cell proliferation of synoviocytes and stops the cell cycle at the G1 phase [92]. miR-338-5p, by inhibiting ADAMTS-9, induced G0/G1 arrest and suppressed RASFs’ biological functions [93,94], whereas miR-152, by targeting ADAM10, suppresses RASF proliferation, reduces the production of TNF-α, IL-1β, IL-6 and IL-8 and supports apoptosis [95]. Sun J. et al. observed that the overexpression of miR-26b inhibits TNF-α, IL-1β and IL-6 expression, and promotes RASF apoptosis [96]. Ju D. et al. reported that the targeting of C-X-C motif chemokine ligand 12 (CXCL 12) by miR-137 significantly inhibits FLS proliferation, migration and invasion, and suppresses the expression of proinflammatory cytokines [97]. miR-140-5p also has an inhibitory role in IL-6 and IL-8 secretion, as well as in RASF proliferation [98]. miR-26b, by targeting signal transducers and activators of transcription (STAT3), reduces the proliferation and expression of proinflammatory cytokines, and induces apoptosis [69]. In contrast, miR-650 promotes the proliferation, migration and invasion of RASFs and, on the other hand, inhibits RASF apoptosis by targeting AKT2 [99]. Ruedel A. et al. observed that miR-188-5p, which is downregulated in RA in vitro and RASF in vivo (RA synovial tissue), directly targets the hyaluronan binding protein KIAA1199 and indirectly regulates COL12A1 and COL1A1. The miR-188-5p expression level correlates with the activation state of RASF and plays a regulatory role in RASF biology. The authors hypothesize that the miR-188-5p-KIAA1199 pathway may be a promising novel therapeutic target in RA [100]. Moreover, the recent study has shown that the main target for miR-192 is caveolin-1, and that this axis inhibits apoptosis [101].

### 3.5. Role of miRNAs in Bone Destruction

**Figure 3:**
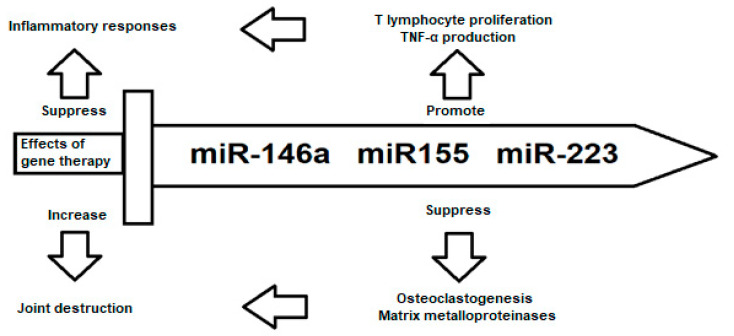
“Double-edged sword”: Opposing roles of miR-146a, miR-155 and miR-223 in the regulation of rheumatoid arthritis (RA) pathogenesis. These miRNAs are positively correlated with disease activation by instigating the inflammatory response. However, at the same time, they have a role in suppressing joint destruction.

Several miRNAs associated with osteoclastogenesis and MMPs are shown in Table 7. RASFs have a major function in joint destruction and are assumed to distribute RA to unaffected joints. miRNA microarray analysis showed that miR-19b is downregulated in activated RASF. The transfection of RASF with miR-19a and miR-19b lowered the levels of MMP-3 and IL-6, indicating that the function of miR-19a and miR-19b is to protect patients with RA from inflammation and joint destruction [80]. Increased levels of miR-146a have been shown to inhibit bone destruction by suppressing osteoclastogenesis [102]. Due to its positive role in inflammatory responses and disease activity, and its negative role in joint destruction, miR-146a acts as a “double-edged sword”, and can be used as a marker for therapy and diagnosis (Figure 3).

The complication of miRNAs is demonstrated by the opposing functions of miR-155 in joint destruction in RA. The overexpression of miR-155 in synovial fibroblasts suppresses the induction of MMP-1 and MMP-3 by Toll-like receptor ligands and cytokines [84]. By contrast, in a K/BxN serum transfer arthritis model, miR-155 insufficiency lowers bone destruction, leading to a smaller generation of osteoclasts [101]. The increased level of miR-203 causes a higher secretion of MMP-1 and IL-6 through the nuclear factor NF-κB pathway, which supports the activated synovial phenotype of RA [103]. miR-223 is another example of the opposing roles for miRNAs in the pathogenesis of RA (Figure 3). In one study, miR-223 was highly expressed in the synovium of RA patients, leading to the in vitro inhibition of osteoclastogenesis [104]. However, another study showed opposing results, where miR-223 led to reduced osteoclastogenesis and bone erosion in a collagen-induced arthritis mouse model [105]. miR-223, along with miR-146a and miR-155, belongs to a group of miRNAs that, on the one hand, have a positive effect on disease activity by stimulating the inflammatory response and, on the other hand, have a negative effect by suppressing joint destruction (Figure 3).

The increased expression of miR-126 has been shown to cause the hypomethylation of CD70 and CD11a genes, and decrease DNA (cytosine-5)-methyltransferase-1 (DNMT1) protein levels, inducing the initiation and improvement of RA [106]. Changed levels of miR-24 also occur in RA, and the upregulation of it is responsible for producing cytokines and inducing the decline of arthritis [49,107].

A greater comprehension of the roles and functions of these miRNAs will allow us to better understand the causes and progression of RA and prepare better therapeutic options [108].

## 4. miRNA—Diagnostic and/or Prognostic Biomarkers for RA?

Rheumatoid arthritis, as a heterogeneous disease with multiple clinical manifestations, has a lack of biomarkers with high selectivity and sensitivity, which is problematic for the prognosis of the disease, as well as for its treatment. Scientists are not only trying to better understand the pathophysiology of arthritis by studying miRNAs, but the future goal is to identify miRNAs as biomarkers in arthritis. The goal is to find new biomarkers that can be used to diagnose and predict a patient’s response to treatment. Recently, miRNAs have emerged as a significant group of new blood-based biomarkers, whose specific expression profile may be associated with disease development and severity, as well as responses to the use of therapy. Moreover, miRNAs can be detected in serum with relatively simple techniques.

Murata et al. have shown that the blood levels of miR-24 and 125-5p were autonomous predictors for RA [107]. The previous study has also shown that higher levels of miR-125b were directly correlated with a higher activity of RA and a good response to treatment with rituximab [109].

Studies by Anaparti V. et al. demonstrated that the miRNA signature in ACPA-positive healthy individuals resembles that of RA ACPA-positive individuals, but differs significantly from that seen in seronegative healthy subjects. Moreover, they also demonstrate that miR-103a-3p is upregulated in RA patients and asymptomatic first-degree relatives compared with healthy subjects, and that miR-103a-3p may be a potential biomarker for predicting disease in individuals at risk for developing RA [55].

Furthermore, Yang S. et al. have shown that miR-221, which is very important in the regulation of cell proliferation, cell cycle, migration and invasion because it regulates target genes, may be engaged in the initiation and progress of RA [90]. They also observed that the inhibition of miR-221 suppressed the expression of proinflammatory cytokines, chemokines, VEGF-A, MMP3 and MMP-9. They hypothesized that miR-221 may be a good potential target in the treatment of RA. Additionally, miR-448, miR-124 and miR-551b could serve as specific biomarkers for distinguishing patients with autoimmune diseases such as RA, systemic lupus erythematosus (SLE), Sjogren’s syndrome (SS) and ulcerative colitis (UC) from healthy subjects [110].

Interestingly, Filkova M. et al. hypothesize that RA severity, disease duration or effect of treatment may modulate the levels of circulating miRNAs in established RA [111]. The authors observed that elevated levels of miR-16 in untreated patients with early RA were associated with better improvement in disease activity over 3 months of follow-up. Moreover, the change in circulating miR-16 levels in the first 3 months after the initiation of therapy correlated with a disease effect in the next 9 months. In addition, they also suggested miR-223 as a biomarker of disease activity in untreated patients with early RA based on significant associations with C-reactive protein (CRP) value and disease activity scale (DAS)-28 scores. Moreover, increased miR-223 levels were linked with better improvements in disease activity in early RA after therapeutic intervention using MTX and/or glucocorticosteroids. They proposed that both miR-16 and miR-223 could be useful in clinical practice to predict the course of the disease, as well as the effectiveness of DMARDs therapy. miR-16-5p, -125b-5p, -223-3p, -23-3p, -126-3p and -146a-5p, which are key players in the pathogenesis of several chronic/autoimmune diseases, such as RA, have the potential to be biomarkers for predicting and monitoring RA therapy. In RA patients who received the anti-TNFα/DMARDs combination therapy, all of these miRNAs were upregulated, leading to the reduction in TNFα, IL-6, IL-17 and CRP value [112]. Another study [113] has shown that miRNA-5196 holds promise as a good marker of anti-TNF-α treatment response in RA patients.

Furthermore, Krintel S.B. et al. observed that miR-22 and miR-886-3p are differentially expressed in RA patients depending on the response to the treatment with adalimumab. The low expression of miR-22 and, at the same time, the higher expression of the miR-886.3p, which increased from 65% to 95%, achieving a EULAR good response to adalimumab (in combination with MTX) in RA patients [114].

In another study, Cunningham C.C. et al. have shown that the serum miRNA signature associated with immune cell dysfunction can be used as a biomarker for RA onset and early progression. Moreover, the identified serum miRNA signature, including miR-126-3p, let-7d-5p, miR-431-3p, miR-221-3p, miR-24-3p and miR-130a-3p, may be useful in the diagnosis of RA in its early stages, as well as in monitoring the therapy response or the risk of the recurrence of the disease in an effective, convenient and cost-effective manner [115].

Guo D. et al. identified three upregulated (miR-187-5p, -4532 and -4516) and eight downregulated (miR-125a-3p, -575, -191-3p, -6865-3p, -197-3p, -6886, -1237-3p and -4436b-5p) miRNAs in the serum of naïve active RA patients. They found that these miRNAs have an important role in the intestinal microbiome dysbiosis, infection, inflammation and immune network through the activation of the T cells, IL-17, TNF, JAK, TLR immune pathways, osteoclast cell differentiation and IgA production [116]. Moreover, Lu H. et al. concluded that both miRNA profiles and microbiomes may have different functions and compositions between patients with undifferentiated arthritis and RA patients. [117].

A study performed by Chen J. et al. showed that miR-486-5p participates in the proliferation, mineralization and differentiation of osteoblasts, leading to the alleviation of RA by Tob1 inhibition, which starts the BMP/Smad signaling pathway [118].

In our earlier study, we confirmed the value of miR-146a and miR-155 as two of the key factors in the early detection of RA, and that miR-26 and miR-155 expose the meaningful diagnostic potential for RA differentiation from healthy individuals [119]. Moreover, we have also shown a correlation between miR-26 and SOCS1, SMAD3 and STAT3, as well as between miR-155 and STAT3, SMAD3 and SMAD4. The most versatile miRNA in RA is miR-155, which not only has the potential to act as a biomarker, but also has great regulatory ability in different cells that target specific miRNAs [83]. It is important to remember that immune cells and inflammatory responses are not exclusive to the pathogenesis of RA, but also take part in the pathogenesis of many autoimmune diseases. miRNAs involved in the pathogenesis of RA will also show positive responses for other diseases, such as OA or psoriatic arthritis (PsA). Because of this, finding the perfect biomarker that is specific for RA diagnosis and treatment is laborious and difficult; however, it is very necessary.

## 5. miRNA-Based Therapy—Present or Future?

In over several years since the detection of the first miRNA, the area of miRNA biology has developed distinctly. Insights into the roles and importance of miRNAs in the development, expansion and progression of diseases, including autoimmune diseases such as RA, have made miRNAs very attractive targets for novel therapeutic approaches. The advancement of miRNA-based therapy is set on the conception that aberrantly expressed miRNAs probably may have a key role in the development of inflammation and autoimmunity. Accordingly, restoring the miRNA function and/or revising the miRNA insufficiency could be used as a novel strategy for inflammatory arthritis treatment [120].

Recently, there was a huge step forward in the field of RNA-based drug molecules. Patisiran was the first RNA interference therapeutic agent, which was accepted as a treatment for hereditary transthyretin amyloidosis (hATTR). Patisiran, by targeting the 3′ untranslated region of transthyretin miRNA, inhibits the hepatic synthesis of transthyretin and revised several clinical manifestations of hATTR [121]. Different antisense oligonucleotide inhibitors that have been approved by the FDA are: mipomersen, which has a role in managing homozygous familial hypercholesterolemia [122], and eteplirsen, which was used for the treatment of Duchenne muscular dystrophy [123].

There are two main branches of miRNA-based therapeutics: mimics and antimiRs (also known as inhibitors of miRNA). Mimics are artificial double-stranded small RNAs that are analogs to the corresponding miRNA sequences. On the other hand, the inhibitors are single stranded, and they target miRNAs [120]. The first miRNA therapeutic drug was locked nucleic acid (LNA) miravirsen. It is a short antisense RNA oligonucleotide complementary to the miR-122 used for the therapy of hepatitis C virus (HCV) infection in phase II of clinical trials. Treatment with miravirsen in monotherapy for four weeks provides long-lasting inhibition of viremia, has a powerful barrier to viral immunity and is properly tolerated in patients with HCV [124]. A Phase IIa clinical trial of miravirsen was conducted in the United States, and also in Poland, Germany, the Netherlands, Slovakia and Romania [125]. However, a recent study showed mutations at the 5′ UTR of HCV RNA in clinical samples (C3A mutation) from patients receiving miravirsen, as well as in in vitro samples (A4C mutation) treated with increasing doses of LNA miravirsen. Although these mutations have been observed in cells or viral relapse patients treated with miravirsen, it is unclear whether these mutations may result in resistance to treatment [120].

Nowadays, about 20 clinical trials have been started (Table 8). They are focused on miRNA- and small interfering RNA (siRNA)-based therapeutics; however, most miRNA therapeutic trials are still in the preclinical stage [125]. siRNA is a powerful tool for silencing target-specific genes via RNAi. Currently, more than 14 RNAi therapeutic programs have entered clinical trials over the past decade. However, only one of them, Excellair, concerns inflammation. Excellair is an anti-inflammatory siRNA that targets and silences the Syk kinase gene, which is a critical factor in inflammatory processes. Excellair has demonstrated its potential and safety in preclinical studies in the treatment of asthma and other inflammatory diseases. This molecule has terminated a phase II clinical trial [125]. The molecules ALN-VSP and bevasiranib, targeting VEGF gene expression, and molecule AGN211745, targeting VEGF receptor I (VEGFRI), may also be used in inflammatory arthritis, where synovial angiogenesis plays an essential role in the early stage of RA by promoting inflammatory processes and joint destruction.

miRNA-based therapy holds many promises for major advances in the therapy of patients with inflammatory arthritis. However, there are still many obstacles before they are approved for clinical use. The main problems of miRNA therapy are safety, efficacy, selectivity and delivery, and when these obstacles are resolved in the coming years, they will become more common in clinical practice [126]. A major challenge in miRNA drug design will be to design a delivery mechanism that would allow greater stability for the therapeutic candidate, and to work on better selectivity to avoid potential toxicities and target misses. Various delivery methods are used to improve bioavailability, such as biodegradable polymers, lipids and PEGylated liposomes [127]. Currently, vesicles with diameters ranging from 50 nm to 500 nm are used for the therapeutic delivery of siRNA and miRNA. miRNAs are not ideal candidates for oral administration. Additionally, their intestinal absorption is not high enough. In general, it is preferred that anti-miR oligonucleotides be administered via the parenteral route, with local delivery, such as by eye or skin, for the therapeutic use of miRNAs, which gives better results in terms of bioavailability [120]. Another challenge may be the innate immune response to a molecule miR-34 that is problematic in clinical applications, which was observed in a clinical trial. In Table 9, the miRNAs and their states of development are summarized.

## 6. Conclusions

Recent studies have shed light on the mechanism of RA and how to better adjust therapy for individual patients. Thanks to the development of biological drugs, the treatment of RA has improved drastically in recent years, with the identification of novel treatments, such as Btk, Syk, PI3K and histone deacetylase (HDAC) inhibitors, providing even more hope for patients with RA. These drugs are in development for clinical use and will result in a shift in RA therapy from what we know today.

The significance of plant-derived miRNA-based targeted therapy in the regulation of inflammation in RA patients was postulated by Saquib M. et al. They demonstrated that the importance of exogenous miRNA for RA is huge, and it would allow more access with less or even minimal side effects. They also noticed that these therapies could be cost effective, with a relatively easy intake with a plant-based diet. The downregulation of disease-associated miRNAs can be reversed by artificially synthesized miRNAs to regain the loss of function. The compatibility of exogenous miRNAs with the human body could be utilized as therapeutic options for the treatment of RA to allow the better targeting of specific immunomodulatory proteins that are involved in the RA pathogenesis [128].

Today, the biggest leap in the understanding, earlier detection and treatment of RA is the role of miRNAs, despite only a few miRNAs taking part in the pathogenesis of RA. In vivo studies proved that these miRNAs have the potential to be used therapeutically [92,129]. One of the major problems with miRNA-based therapies is the delivery system. miRNA delivery systems should be target-specific and should be capable of delivering drugs to target cells or tissues. In addition, the synchronized delivery of miRNA-based therapies and the conventional therapies used in RA or other autoimmune diseases will be also highly challenging. Nevertheless, miRNA-based therapeutics will overcome these difficulties and will enter the clinic as next-generation drugs. miRNAs, as a novelty therapeutic option, definitely have the potential to contribute significantly to the future of medicine.

Research into small RNAs (also known as noncoding RNAs), which include miRNAs, is still in its infancy. In recent years, scientists from around the world have started paying more attention to miRNAs. Being able to understand the cross-talk between miRNAs and other noncoding RNAs will allow us to better understand the gene regulatory networks implicated in the pathogenesis of RA. The better identification and understanding of the expression patterns of specific miRNAs in RA, as well as the role of these miRNAs in pathogenesis, would allow for a novel therapeutic approach for RA, as well as for other types of autoimmune and inflammatory arthritis, and help to identify new molecular markers for the earlier diagnosis of patients who are at risk [130].

## Figures and Tables

**Table 1:** List of increased levels of miRNA in PBMCs with their targeted genes [8].

Increased Level of miRNA	Targets	Subject of Studies
miR-16	Unknown	Human
miR-103a	AGO2, TP53	Human
miR-132	Unknown	Human
miR-145	Unknown	Human
miR-146a *	IRAK1, TRAF6	Human
miR-155 *	APAF1, CASP10, SHIP-1, SOCS1,	Human and Murine
miR-221	Unknown	Human
miR-222	Unknown	Human
miR301a	PIAS3	Human

Abbreviations: AGO: Protein argonaute-2, APAF1: Apoptotic protease activating factor 1, CASP10: Caspase 10, IRAK1: Interleukin-1 receptor-associated kinase 1, JNK: c-Jun N-terminal kinase, PIAS3: Protein inhibitor of activated STAT3, SHIP-1: Src homology 2-containing inositol phosphatase-1, SOCS1: Suppressor of cytokine signaling 1, TP53: Tumor protein p53, TRAF6: Tumor necrosis factor receptor-associated factor 6. * Are mentioned in more than one study.

**Table 2:** List of decreased levels of miRNA in PBMCs with their targeted genes [8].

Decreased Level of miRNA	Targets	Subject of Studies
let-7a	ERK1, ERK2, K-Ras, JNK	Human
miR-21	Foxp3, STAT3, STAT5	Human
miR-125b	Unknown	Human
miR-548	NF-κB pathway, TLR-4	Human

Abbreviations: ERK1/2: Extracellular signal-regulated kinase ½, Foxp3: Forkhead box P3, K-Ras: Kirsten rat sarcoma virus gene, NF-κB: Nuclear factor kappa-beta, TLR: Toll-like Receptor.

**Table 3:** List of increased levels of miRNA in RASFs with their targeted genes [8].

Increased Level of miRNA	Targets	Subject of Studies
miR-18a	TNFAIP-3	Human
miR-19 *	TLR2 pathway	Human
miR-21	NF-κB pathway	Murine
miR-125b	NF-κB pathway	Human
miR-126 *	PIK3R2	Human
miR-143	IGF1R, IGFBP5, Ras MAPK, p38 MAPK	Human
miR-145	SEMA3A	Human
miR-146a *	IRAK-1, TRAF6	Human and Murine
miR-155 *	IKBKE, JAK2, STAT3	Human and Murine
miR-203 *	NF-κB pathway	Human
Unknown change to miR-218	ROBO1, Wnt/β-catenin	Human
miR-221 *	Wnt, BMP	Human and Murine
miR-222	Wnt/cadherin	Murine
miR-223 *	IL-17RD, NFI-A	Human and Murine
miR-323	Wnt/cadherin	Murine
miR-338 *	NFAT5	Human
miR-346	Btk, TTP	Human
miR-522	SOCS3	Human
miR-663	APC	Human

Abbreviations: APC: Adenomatous polyposis coli gene, Btk: Bruton’s tyrosine kinase, IL-17RD: Interleukin 17 receptor D, IRAK: Interleukin-1 receptor-associated kinase, JAK: Janus kinase, NFAT5: Nuclear factor of activated T cells 5, NFI-A: Nuclear factor 1-A, NF-κB: Nuclear factor kappa-beta, PIK3R2: Phosphatidylinositol 3-kinase regulatory subunit 2, p38 MAPK: Mitogen-activated protein kinase, Ras MAPK:, ROBO1: Roundabout 1, SCDF1: Stromal cell-derived factor 1, Sirtuin1:, SEMA3A: Semaphorin-3a, SOCS3: Suppressor of cytokine signaling, STAT: Signal transducer and activator of transcription, TLR2: Toll-like receptor, TNFAIP-3: Tumor necrosis factor a-induced protein 3, TTP: Tristetraprolin, Wnt: Wingless/integrated, Wnt/β-catenin: Wingless/integrated/β-catenin, Wnt/cadherin: Wingless/integrated/cadherin. * Are mentioned in more than one study.

**Table 4:** List of decreased levels of miRNA in RASFs with their targeted genes [8].

Decreased Level of miRNA	Targets	Subject of Studies
miR-10b *	BTRC, IRAK4, TAK1, TBX5	Human
miR-17	TRAF2	Human
miR-20a *	ASK1, TXNIP	Human and Murine
miR-22	Cyr61	Human
miR-23b	IKK- α, TAB2, TAB3	Human and Murine
miR27a	FSTL1, NF-κB pathway, TR4 pathway	Human
miR-29a *	STAT3	Human
miR-30-3p	BAFF	Human
miR-34 *	XIAP	Human
miR-124a *	CDK2, MCP1	Human
miR-137	CXCL12	Murine
miR-140-5p	SCDF1, Sirtuin1	Human
miR-152 *	ADAM10, DNMT1	Human and Murine
miR-188-5p	CEMIP	Human
miR-192	Caveolin 1	Human
miR-199a	RB1	Human
miR-204	ATF2	Human
miR-211	ATF2	Human
miR-212	SOX5	Human
miR-375	Wnt/FZD8	Murine
miR-539	OPN	Human
miR-650	AKT2	Human

Abbreviations: ADAM10: A disintegrin and metalloproteinase domain-containing protein 10, AKT2: Protein kinase B 2, ASK1: Apoptosis signal-regulating kinase 1, ATF2: Activating transcription factor 2, BAFF: B cell-activating factor, Caveolin 1:, CDK2: Cyclin-dependent kinase 2, CEMIP: Cell migration-inducing and hyaluronan-binding protein, Cyr61: Cysteine-rich angiogenic inducer 61, CXCL12: C-X-C motif chemokine ligand 12, DNMT1: DNA (cytosine-5)-methyltransferase-1, FSTL1: Follistatin-like protein 1, IKK- α: IkB kinase a, MCP1: Monocyte chemoattractant protein 1, NF-κB: Nuclear factor kappa-beta, OPN:, SCDF1: Stromal cell-derived factor 1, Sirtuin1:, STAT: Signal transducer and activator of transcription, TAB: TAK1/MAP3K7 binding protein, TAK1: Transforming growth factor-beta-activated kinase 1, TBX5: T-box transcription factor 5, TRAF: Tumor necrosis factor receptor-associated factor, TR4 pathway:, Wnt: Wingless/integrated, Xiap: X-linked inhibitor of apoptosis protein. * Are mentioned in more than one study.

**Table 5:** Role of microRNAs (miRNAs) in immune and inflammatory responses in rheumatoid arthritis (RA) [8,87].

miRNAs	Regulation of T Lymphocytes	Inflammatory Response
Let-7a	Positive regulator	
miR-16	Positive regulator	
miR-17		Negative regulator
miR-18		Positive regulator
miR-19a/b		Negative regulator
miR-20		Negative regulator
miR-21	Positive regulator	
miR-26	Positive regulator	
miR-125b		Positive regulator
miR-132	Positive regulator	
miR-146a	Positive regulator	Positive regulator
miR-146b	Positive regulator	
miR-150	Positive regulator	
miR-155	Positive regulator	Positive regulator
miR-203		Positive regulator
miR-223		Positive regulator
miR-323-3p	Positive regulator	Positive regulator
miR-451		Negative regulator

**Table 6:** Role of microRNAs (miRNAs) in synovial hyperplasia in rheumatoid arthritis (RA) [8,87].

miRNAs	Apoptosis	Cellular Proliferation	Migration
miR-15a	Positive regulator		
miR-26b	Positive regulator	Negative regulator	
miR-29a	Positive regulator	Negative regulator	
miR-34a	Positive regulator		
miR-124a		Negative regulator	
miR-126	Negative regulator	Positive regulator	
miR-137		Negative regulator	Negative regulator
miR-140-5p		Negative regulator	
miR-152	Positive regulator	Negative regulator	Negative regulator
miR-188-5p			Negative regulator
miR-192	Positive regulator	Negative regulator	
miR-221		Negative regulator	Negative regulator
miR-338-5p	Positive regulator	Negative regulator	Negative regulator
miR-650	Negative regulator	Positive regulator	Positive regulator

**Table 7:** Role of microRNAs (miRNAs) in bone destruction in rheumatoid arthritis (RA) [8,87].

miRNAs	Osteoclast Generation	Matrix Metalloproteinases
miR-19a/b		Negative regulator
miR-106b	Positive regulator	
miR-146a		Negative regulator
miR-155	Positive regulator	Negative regulator
miR-203		Positive regulator
miR-223	Positive/negative regulator	

**Table 8:** Therapeutics siRNAs that are in a development phase, and their indication [125].

Therapeutic siRNAs	Target	Indication	Phase
AGN21174	VEGFR1 gene	Age-related macular leukemia	Terminated in phase II
AGN211745	VEGFR1 gene	Treatment of age-related macular degeneration	Clinical trial phase II
ALN-RSV01	RSV nucleocapsid	Treatment of RSV infection during lung transplantation	Clinical trial phase IIb
ALN-TTR02	TTR	Treatment of transthyretin-mediated amyloidosis	APOLLO study phase III
ALN-VSP	VEGF gene	Treatment of liver cancer	Completed phase I
ApoB SNALP	Apolipoprotein B gene	Treatment of hypercholesterolemia	Concluded clinical trial phase I
Atu-027	Protein kinase N3 gene	Treatment of advanced solid tumors	Clinical trial phase I
Bevasiranib	VEGF gene	Treatment of AMD or diabetic macular edema	Clinical trial phase III
CALAA-01	M2 subunit of ribonucleotide reductase	Inhibit tumor and cancer therapy	Clinical trial phase Ib
Excellair	Syk gene	Treatment of inflammatory disorders	Clinical trial phase II
QPI-1002	p53	Avoidance of AKI, prophylaxis of DGF	In phase II obtained Orphan drug designation
QPI-1007	Caspase-2 gene	Treatment of nonarthritic anterior ischemic optic neuropathy	
PF-04523655	HIF-1-responsive gene, RTP801	Treatment of age-related macular degeneration and diabetic macular edema	Clinical trial phase II
PF-655	RTP801 gene	Treatment of age-related macular degeneration	Clinical trial phase II
RXI-109	CTGF gene	Treatment of fibrosis and ocular disorders	Clinical trial phase I
SYL040012	β_2_-adrenergic receptor gene	Ocular hypertension	Completed phase II

CTGF: Connective tissue growth factor, DGF: Delayed graft function, RSV: Respiratory syncytial virus, Syk: Spleen tyrosine kinase, TTR: Transthyretin.

**Table 9:** Therapeutics miRNAs that are in a development phase, and their indication [125].

Therapeutic miRNAs	Target	Indication	Phase
MGN-1374	miR-15 and miR-195	Treatment of post-myocardial infraction remodeling	Preclinical stage
MGN-2677	miR-143/145	Treatment of vascular disease	Being prepared
MGN-4220	miR-29	Treatment of cardiac fibrosis	Being prepared
MGN-4893	miR-451	Treatment of disorders (polycythemia vera)	Being prepared
MGN-5804	miR-378	Treatment of cardio metabolic disease	Being prepared
MGN-6114	miR-92	Treatment of peripheral arterial disease	Being prepared
MGN-9103	miR-208	Treatment of chronic heart failure	Being prepared
Miravirsen		HCV infection	Clinical trial phase IIa
MiRX34		Treatment of variety of cancers	Stopped in clinical trial phase I
RG-012		Treatment of Alport syndrome	Being prepared for clinical trial phase II
RG-101	GalNAc-conjugated anti-miR	Treatment of HCV	

HCV: Hepatitis C virus.

## Data Availability

Not applicable.

## Abbreviations

ACPA	Anti-citrullinated protein antibody
ADA	Adalimumab
ADAMTS	A disintegrin and metalloproteinase with thrombospondin motifs
Bcl-2	B cell lymphoma 2
bDMARDs	Biological disease-modifying anti-rheumatic drugs
Btk	Bruton’s tyrosine kinase
CRP	C-reactive protein
csDMARDs	Conventional synthetic disease-modifying anti-rheumatic drugs
CTLA-4	Cytotoxic T-lymphocyte-associated protein 4
CXCL 12	C-X-C motif chemokine ligand 12
DAS	Disease activity scale
DNMT1	DNA (cytosine-5)-methyltransferase-1
DMARDs	Disease-modifying anti-rheumatic drugs
ETA	Etanercept
Foxp3	Forkhead transcription factor
GM-CSF	Granulated macrophage colony-stimulating factor
GOL	Golimumab
hATTR	Hereditary transthyretin amyloidosis
HCV	Hepatitis C virus
HDAC	Histone deacetylase
HIF1α	Hypoxia-inducible factor 1α
IFX	Infliximab
ILs	Interleukins
JAK	Janus kinase
LNA	Locked nucleic acid
M-CSF	Macrophage colony-stimulating factor
miRNA	MicroRNA
MMPs	Matrix metalloproteinases
MSC	Mesenchymal stem cells
MTX	Methotrexate
NF-κB	Nuclear factor kappa-beta
OA	Osteoarthritis
PBMC	Peripheral blood mononuclear cell
PI3K	Phosphoinositide 3-kinase
PIK3R2	Phosphatidylinositol 3-kinase regulatory subunit 2
PsA	Psoriatic arthritis
qRT-PCR	Quantitative reverse transcription–polymerase chain reaction
RA	Rheumatoid arthritis
RANKL	Receptor activator of nuclear factor-kappa B ligand
RASF	Rheumatoid arthritis synovial fibroblast
RF	Rheumatoid factor
RORC2	Retinoic acid receptor-related orphan receptor variant 2
RTX	Rituximab
siRNA	Small interfering RNA
SLE	Systemic lupus erythematosus
SS	Sjogren’s syndrome
STAT3	Signal transducers and activators of transcription
Syk	Spleen tyrosine kinase
TCZ	Tocilizumab
TGF-β	Transforming growth factor-β
Th cells	T helper cells, helper T cells
TIMPs	Tissue inhibitors of metalloproteinase
TNF	Tumor necrosis factor
TLR	Toll-like receptor 2
Treg cells	T regulatory cells, regulatory T cells
tsDMARDS	Targeted synthetic disease-modifying anti-rheumatic drugs
Tyk2	Tyrosine kinase 2
UC	Ulcerative colitis
VEGF	Vascular endothelial growth factor
VEGFRI	VEGF receptor I
Wnt	Wingless/integrated
XIAP	X-linked inhibitor of apoptosis protein
